# First National Data Reporting Nursing Home Social Services Directors’ Training Interests and Needs

**DOI:** 10.1093/geroni/igaa057.2533

**Published:** 2020-12-16

**Authors:** Mercedes Bern-Klug, Elizabeth Cordes

**Affiliations:** University of Iowa, Iowa City, Iowa, United States

## Abstract

A national sample of 924 social services directors reported training needs in three ways. First, their level of interest in 14 topics, second by how much preparation time needed to provide one-on-one training to a colleague about 27 difference issues/tasks (no time needed, up to 2 hours, up to 10 hours, not able to do), and third, by indicating their top training priority. At least 2/3s reported interest in each of the 14 topics with 86% interested in common mental health and psychosocial challenges, and 86% in the psychosocial needs of persons with dementia. Education, social work licensure, and characteristics of the nursing home explained some of the variation, e.g. respondents with a social work degree reported higher interest in more training in trauma-informed care and culturally competent care. Dementia was by far the highest training priority, followed by better understanding of regulations, behavioral health issues, and trauma-informed care.

